# Associations among Elder Abuse, Depression and PTSD in South Korean Older Adults

**DOI:** 10.3390/ijerph15091948

**Published:** 2018-09-06

**Authors:** Yun-Jung Choi, Meaghan O’Donnell, Hwa-Bok Choi, Hae-Sun Jung, Sean Cowlishaw

**Affiliations:** 1 Red Cross College of Nursing, Chung-Ang University, Seoul 06974, Korea; ghkqhr83@naver.com (H.-B.C.); haesun710@hanmail.net (H.-S.J.); 2 Phoenix Australia Centre for Posttraumatic Mental Health, Department of Psychiatry, University of Melbourne, Melbourne, VIC 3053, Australia; sean.cowlishaw@unimelb.edu.au; 3 Bristol Medical School, University of Bristol, Bristol BS8 2PS, UK

**Keywords:** depression, elder abuse, mistreatment, older adults, PTSD, senior citizens

## Abstract

Increasing attention is being placed on the prevalence of elder abuse and its impact on mental health. This study conducted a survey of 172 elderly people in South Korea to determine the prevalence of elder abuse and the relationships involving elder abuse, depression and posttraumatic stress disorder (PTSD). Participants completed a battery of self-report questionnaires, which included the Korean Geriatric Depression Screening Scale (KGDS) and Impact of Event Scale-Revised Korean version (IES-R-K). Descriptive analyses were conducted to examine the frequency of specific forms of abuse. Logistic regression models were estimated to identify the factors that contributed to risk of abuse exposure and the relationship between exposure and PTSD or depression. The results indicated around 22% of the participants reported abuse exposure, which most commonly included being refused physical contact, verbal threats, and/or being excluded from decision-making about personal issues. Low education and being unmarried, separated or divorced was associated with an increased risk of abuse exposure. There were strong associations between elder abuse and PTSD symptoms, while comparable relationships with depression were weaker and were not robust to the inclusion of control variables. The findings provided empirical support for the relationship between abuse experiences of the elderly and poor mental health and raise important issues for the mental health care of the elderly.

## 1. Introduction

An estimated 5 to 10% of older people in the United States experience mistreatment each year [1]. In a meta-analysis of data from 59,203 older adults across 44 studies, the mean prevalence rate of elder abuse was 15.7% [2], with a range between 3.2% and 27.5% [3]. Elder abuse is defined as a single or repeated act, occurring within any relationship where there is an expectation of trust, which causes harm or distress to an older person over the age of 65. It includes a range of types of abuse including physical, sexual, emotional and financial abuse, as well as abandonment and neglect [4]. 

Prevalence rates differ depending on the type of elder abuse. In a South Korean survey of elder abuse among older adults, emotional abuse was highest at 40.1 %, followed by physical abuse at 31.3%, neglect at 19.1%, financial abuse at 7.2%, sexual abuse at 1.3% and abandonment at 1.0% [5]. Globally, rates of abuse are 11.6% for psychological abuse, 6.8% for financial abuse, 4.2% for neglect, 2.5% for physical abuse and 0.9% for sexual abuse [4]. Generally, emotional abuse occurs more frequently than physical abuse. In order to minimize elder abuse, South Korea has developed a national institution for the protection of the elderly, which is implementing preventive projects in conjunction with local government. Despite this, the number of reported incidents of elder abuse reports has risen sharply each year, reaching 10,569 in 2014, an increase of 40.8% over 2010 [5].

Experiences of elder abuse are associated with symptoms of depression [6,7]. Elder abuse survivors with depression are also at increased risk for suicidal thoughts [8]. Posttraumatic stress disorder (PTSD) is a psychiatric disorder featuring symptoms of intrusion, avoidance, negative alterations in cognition and mood and hyper-arousal. Exposure to a traumatic event such as elder abuse may be connected with the development of PTSD [9,10].

Depression and PTSD are often comorbid. Severe depressive symptoms were comorbid with PTSD symptoms in more than one-third of the people who experienced traumatic events [11] and past incidence of depression was strongly associated with probable PTSD [10]. While there have been several studies investigating the relationship between elder abuse and depression [6,7,8], to date, no study has examined the relationship between elder abuse and PTSD [10]. 

The broad purposes of this study were to help inform programs for improving the identification and management of depression and PTSD in abused senior citizens. Specific aims of the study were to:

(1) Examine the frequency of specific forms of elder abuse in a community dwelling sample of adults from South Korea;
(2) Identify risk factors for elder abuse; and
(3) Evaluate the mental health implications of elder abuse, with a particular focus on depression and PTSD.

## 2. Methods

### 2.1. Participants

Participants were community-residing adults aged 65 and older who could communicate with the investigator. They were recruited by convenience sampling via advertisements from community academies for the elderly and community mental health improvement centers, located in Seoul and Gyeonggi-Province, South Korea. People were excluded if cognitive function was low, as measured by the MMSE (Mini-Mental State Examination; less than 24 points) [6]. The participants were reimbursed for their participation in the study with grocery items. The criteria for inclusion were the following:

(1) Older adults aged 65 and older who scored more than 24 points on the MMSE.
(2) Older adults who understood the purpose of the study and voluntarily signed the consent form.
(3) Older adults who could read and respond to the self-report questions of the research instruments.

### 2.2. Instruments

#### 2.2.1. Korean Elder Abuse Scale

The experience of elder abuse was measured using the assessment tool of the South Korea Elder Protection Agency [5]. The questionnaire contains 20 items which are shown in Table 2 and addresses six domains: (a) physical abuse, (b) emotional abuse, (c) sexual abuse, (d) financial abuse, (e) neglect and (f) abandonment. Each item was scored on a nominal scale, on which participants chose either yes or no based on their lifetime experiences. The Cronbach’s alpha for this scale was 0.89 [8].

#### 2.2.2. Korean Geriatric Depression Screening Scale (KGDS)

The level of depression in the elderly was evaluated with the KGDS, adapted for South Korean older adults from the GDS [12]. The final version of the KGDS has improved discriminatory diagnostic power, approximately 10% greater than the GDS, and shows sufficient reliability and validity. The scale consists of 30 items, including 16 negative and 14 positive items. Participants answer yes or no depending on their level of depression. The 16 negative items were scored 1 point for yes and 0 points for no and the 14 positive items were scored as 1 point for no and 0 for yes. The total score ranged from 0 to 30. More than 22 points indicates severe depression, while scores of 19 to 21 indicate moderate depression, 14 to 18 indicates mild depression, and scores of 13 or less is considered normal. The Cronbach’s alpha for this scale was 0.88 and the split-half reliability was 0.79 [13].

#### 2.2.3. Impact of Event Scale-Revised Korean Version (IES-R-K)

The level of PTSD was examined using the IES-R-K, which was modified for Korean people from IES-R [14]. This scale, which is a useful self-rating diagnostic instrument for PTSD symptoms, comprises 22 items [15]. Each item was rated on a Likert scale ranging from 0 to 4 points; higher scores indicate a higher level of PTSD. The total score ranges from 0 to 88. A score of 25 or more was taken as the cutoff for the high-risk group, 18 to 24 points for the risk group and less than 18 points for the normal group. The split-half reliability for this scale was 0.71 and test-retest reliability was 0.89 [15].

#### 2.2.4. Demographic Characteristics

The general characteristics of participants were assessed including gender, age, educational level, family composition and marital status. In addition, the participants’ occupation, chronic disease, subjective health and subjective financial status were also assessed.

### 2.3. Data Collection

Data were collected from community academies for the elderly and community mental health improvement centers. The researcher visited each organization in person and conducted a survey of older adults who voluntarily agreed to participate. The researcher filled out the questionnaire instead of the participants when they were physically inconvenienced and asked the agency’s staff to participate together in the survey for the protection of the elderly. There were 175 research questionnaires distributed across the four organizations and all were returned; however, 3 were excluded because of incomplete responses and thus a total of 172 were included in the analyses.

### 2.4. Ethical Considerations

All participants voluntarily signed the consent form after the purpose and procedures of the study were explained. Participants were advised that they could withdraw from the survey at any time without disadvantage and that their data would be used only for research purpose. This study received institutional review board approval (No. 1041078-201703-HRSB-047-01).

### 2.5. Data Analysis

Data file preparation was conducted in SPSS version 24, while subsequent analyses were conducted in Program R version 3.4.3. These initially comprised descriptive analyses of items from the Korean Elder Protection Agency Assessment Tool, which were intended to establish the frequency of exposure to specific types of elder abuse. This included an aggregate measure of exposure to any form of abuse, which was treated as a dependent variable in a subsequent series of logistic regression models which examined potential risk factors including socio-demographic characteristics (e.g., gender, age, family composition) and financial or health-related characteristics (e.g., subjectively rated health status, subjectively rated financial status), in order to establish bivariate associations and sub-groups that were distinctively vulnerable to elder abuse. Finally, logistic regression models were also estimated to evaluate the mental health implications of elder abuse. These models specified elder abuse as an explanatory variable and examined associations with mental health outcomes including binary measures of depression (mild/moderate/severe) and PTSD (high risk/risk). Unadjusted models were specified initially to quantify bivariate associations, while adjusted models including additional sociodemographic predictors were presented in order to examine potential confounding from control variables. Estimates of the Odds Ratio (OR) and 95% Confidence Interval (CI) derived from these models were reported as an index of effect size and thus the magnitude of associations. By way of illustration, OR estimates of 4.0 and 5.0, respectively, would suggest that predictor variables are associated with a 4-fold or 5-fold increase in the odds of the relevant outcome (e.g., elder abuse).

## 3. Results

### 3.1. General Characteristics of Participants

Of the participants, 69.2% were women and 30.8% were men. The majority of participants (56.4%) were in their 70s and 21.5% were in their 80s. As for education levels, 36.0% of the respondents graduated from high school or above and 34.3% from middle school. A total of 57% of the participants lived with their spouses, 22.7% lived with their children and 19.8% lived alone. A total of 66.3% of the respondents were married, 24.4% were widowed and 6% were unmarried. The majority of the participants were not employed (73.8%). A total of 57% of the respondents reported being diagnosed with a chronic disease (Table 1).

### 3.2. Descriptive Analyses

Table 2 presents the frequencies of items from the Korean Elder Protection Agency Assessment Tool, which indicates that around 22% of participants reported any form of elder abuse. The most common form of abuse involved emotional abuse, such as being refused physical contact, being subject to verbal threats and being excluded from decision-making about personal issues. Other forms of abuse included violations of their legal rights of personal property and limiting social relations. Theft of income, property and wages were reported less often (by 3–4% of the sample). There were infrequent reports of various other forms of abuse, including physical or sexual assault and threats of physical harm. 

To examine the predictors of abuse, a series of bivariate logistic regression models were conducted (see Table 3). As can be seen, there were no significant associations with elder abuse and age, gender, subjectively rated health or financial status. However, there were significant effects for: (a) education, with participants who completed high school or above indicating reduced risk of abuse, relative to those who completed primary school or less; and (b) marital status, with participants who were unmarried, separated or divorced indicating higher rates of elder abuse, relative to those who were married. 

To examine the relationship between any elder abuse and PTSD/depression, further logistic regression models were run (Table 4). These included both unadjusted (bivariate) models, as well as adjusted models which included simultaneous controls for the socio-demographic and subjective measures listed in Table 2. As can be seen, there was significant bivariate associations between elder abuse exposure and depression, which was slightly reduced and non-significant when controlling for other variables. In contrast, the association with elder abuse and PTSD was substantially stronger and was also robust to the inclusion of covariates. Results from the adjusted model indicated that participants reporting any exposure to elder abuse also exhibited a near 5-fold increase in the odds of PTSD scores that were above cut-off criteria.

## 4. Discussion

This study investigated the prevalence of elder abuse and its relationship to depression and PTSD. The demographic characteristics of this sample were similar to a national representative survey of South Korean older adults (*n* = 2362) [16], which may speak to the generalizability of the findings in this current study. 

Consistent with other South Korean studies [5], emotional abuse was the most common form of abuse reported by older people. This is consistent with a WHO report which also reported the high rates of emotional abuse in elderly people [4]. It may be that interventions designed to educate care givers and support services about what constitutes emotional abuse would help to mitigate this risk.

The current study showed a significant correlation between abuse experience and depression, and thus replicated studies that have also reported relationships between depression and abuse in the elderly [6,7]. However, we also extended the findings of other studies by considering the impacts of demographic variables on this relationship. Importantly, we found the relationship between depression and abuse became non-significant when considering the impact of gender, age, education, marital status, subjective health and financial status. While other studies have found a relationship between depression and elder abuse, few have taken into account the impact of demographic characteristics. This suggests the relationship between depression and elder abuse is complex and large, longitudinal studies are required to explore this relationship further. 

Previous studies have shown that exposure to trauma increases the risk of PTSD [10]. In this study, there was a strong relationship between elder abuse and PTSD and this relationship remained significant after demographic factors were taken into account. This is an important funding and highlights the need to assess both PTSD symptoms and the potential for elder abuse in older people. 

Elder abuse is underrepresented in the scientific literature. Over 20% of our sample reported experiencing some form of abuse. While acknowledging that the current study was limited in terms of the size and self-selected nature of the sample, it does point to the fact that elder abuse is an important focus for future research. This should investigate the types and frequencies of different types of elder abuse and their impact on mental health. Future research should also focus on the treatment of depression and PTSD after elder abuse.

There current study was characterized by a number of limitations. For example, all measures were based on self-report and do not necessarily indicate diagnoses of major depression or PTSD. The sample size was small and was unable to support complex analyses involving large numbers of explanatory variables. Furthermore, the sample was not derived using systematic sample techniques and is not fully representative of community dwelling older adults in South Korea. This is a cross-sectional study and causation can be inferred but not proven.

## 5. Conclusions

In addition to physical injuries, older adults who are subjected to elder abuse experience emotional difficulties such as depression and PTSD. It is necessary to develop effective abuse management programs that aim to increase understanding of elder abuse and improve strategies for dealing with depression and PTSD in the elderly.

## Figures and Tables

**Table 1:** General characteristics of the participants.

Variables	Categories	*n*	%
Gender	Male	53	30.8
	Female	119	69.2
Age (year)	65~69	36	20.9
	70~79	97	56.4
	80~89	37	21.5
	90~	2	1.2
Education level	Uneducated	8	4.7
	Elementary school	43	25.0
	Middle school	59	34.3
	High school or above	62	36.0
Family composition	Elderly couples	98	57.0
	Single elderly	34	19.8
	Living with adult children	39	22.7
	Others	1	0.6
Marital status	Married	114	66.3
	Married (spouse death)	42	24.4
	Married (separation/divorce)	5	2.9
	Unmarried	11	6.4
Employment status	Yes	45	26.2
	No	127	73.8
Chronic disease	Yes	98	57.0
	No	74	43.0
Subjective health status	Good	45	26.2
	Normal	102	59.3
	Poor	25	14.5
Subjective financial status	Good	46	25.6
	Normal	114	66.3
	Poor	14	8.1

Note: Percentages may not add up to exactly 100 per cent, owing to rounding off.

**Table 2:** Item-level frequencies and proportions with 95% Confidence Intervals (CIs) for elder abuse indicators.

Domain	Item	*n*	%	95% CI	
				LB	UB
Physical abuse	Physically assaulted	1	0.6	0.0	3.2
	Forced into confined space and locked in	0	0.0	-	-
	Forced to be physically restrained	2	1.2	0.1	4.1
	Threatened with a high risk of physical harm	2	1.2	0.1	4.1
	Subjected to life-threatening physical attacks	4	2.3	0.6	5.8
	Restricted or hindered by medication	1	0.6	0.0	3.2
	Forced to do things I did not want or difficult to perform	1	0.6	0.0	3.2
	Any physical abuse	5	2.9	1.0	6.7
Sexual abuse	Sexually abused	0	0.0	-	-
	Received expressions or actions of sexual insult	3	1.7	0.4	5.0
	Any sexual abuse	3	1.7	0.4	5.0
Emotional abuse	Refused physical contact	17	9.9	5.9	15.4
	Limiting social relations	6	3.5	1.3	7.4
	Subjected to verbal threats	9	5.2	2.4	9.7
	Excluded from decision-making about personal issues	9	5.2	2.4	9.7
	Any emotional abuse	31	18.0	12.6	24.6
Financial abuse	Theft of income, property or wages	6	3.5	1.3	7.4
	Violate the legal rights of personal property	3	1.7	0.4	5.0
	Not allowed to make decisions about my property use or management	7	4.1	1.7	8.2
	Any financial abuse	11	6.4	3.2	11.2
Neglect	Have not received basic support for daily living, such as clothes, food, or housing	4	2.3	0.6	5.8
	Have not received financial support to maintain my basic living	2	1.2	0.1	4.1
	Have not received medical support though having health problems	2	1.2	0.1	4.1
	Any neglect	6	3.5	1.3	7.4
Abandonment	Told to be sent out from my home	3	1.7	0.4	5.0
	Any abandonment	3	1.7	0.4	5.0
**Any abuse**		**37**	**21.5**	**15.6**	**28.4**

**Table 3:** Frequencies and Odds Ratios (ORs) from bivariate Logistic Regression models predicting any abuse.

Variables	Any Abuse		OR	95% CI	
	*n*	%		LB	UB
Gender (ref: Female)	28	23.5			
Male	9	17.0	0.66	0.28	1.48
Age (ref: 65–69 years)	6	16.7			
70–79 years	21	21.6	1.38	0.53	4.06
80+ years	10	25.6	1.72	0.57	5.64
Education (ref: Elementary school or less)	16	31.4			
Middle school	12	20.3	0.56	0.23	1.32
High school or above	9	14.5	0.37 *	0.14	0.92
Family composition (ref: Couple)	21	21.4			
Single	8	23.5	1.14	0.43	2.82
Living with adult children	8	20.5	0.96	0.37	2.33
Marital status (ref: Married)	22	19.3			
Widow(er)	7	16.7	0.84	0.31	2.05
Unmarried/separated/divorced	8	50.0	4.18 **	1.40	12.61
Self-rated health (ref: Normal)	19	18.6			
Good	10	22.2	1.25	0.51	2.91
Poor	8	32.0	2.06	0.75	5.38
Chronic disease (ref: Yes)	16	21.6			
No	21	21.4	1.01	0.48	2.10
Financial status (ref: Normal)	19	18.6			
Good	10	22.2	1.39	0.59	3.14
Poor	8	32.0	1.67	0.43	5.53

NB: * *p* < 0.05, ** *p* < 0.01

**Table 4:** Descriptive statistics and Odds Ratios (ORs) from Logistic Regression models predicting Depression and Posttraumatic stress disorder (PTSD) categories.

Outcome	No Abuse		Any Abuse		Logistic Regression Models					
					Unadjusted			Adjusted		
	*n*	%	*n*	%	OR	95% CI		aOR	95% CI	
						LB	UB		LB	UB
Depression (mild/moderate/severe)	50	37.0	21	56.8	2.23 *	1.07	4.73	1.93	0.79	4.80
PTSD (moderate/severe)	38	28.1	23	62.2	4.19 ***	1.98	9.18	4.95 ***	2.13	12.10

NB: * *p* < 0.05, *** *p* < 0.001. Adjusted models include gender, age, education, marital status, subjective health and financial status as covariates.
